# Tumors diagnosed as cerebellar glioblastoma comprise distinct molecular entities

**DOI:** 10.1186/s40478-019-0801-8

**Published:** 2019-10-28

**Authors:** Annekathrin Reinhardt, Damian Stichel, Daniel Schrimpf, Christian Koelsche, Annika K. Wefers, Azadeh Ebrahimi, Philipp Sievers, Kristin Huang, M. Belén Casalini, Francisco Fernández-Klett, Abigail Suwala, Michael Weller, Dorothee Gramatzki, Joerg Felsberg, Guido Reifenberger, Albert Becker, Volkmar H. Hans, Marco Prinz, Ori Staszewski, Till Acker, Hildegard Dohmen, Christian Hartmann, Werner Paulus, Katharina Heß, Benjamin Brokinkel, Jens Schittenhelm, Rolf Buslei, Martina Deckert, Christian Mawrin, Ekkehard Hewer, Ute Pohl, Zane Jaunmuktane, Sebastian Brandner, Andreas Unterberg, Daniel Hänggi, Michael Platten, Stefan M. Pfister, Wolfgang Wick, Christel Herold-Mende, Andrey Korshunov, David E. Reuss, Felix Sahm, David T. W. Jones, David Capper, Andreas von Deimling

**Affiliations:** 1 Department of Neuropathology, University Hospital Heidelberg, Im Neuenheimer Feld 224, 69120 Heidelberg, Germany; 2 Clinical Cooperation Unit Neuropathology, German Cancer Consortium (DKTK), German Cancer Research Center (DKFZ), Heidelberg, Germany; 3 Department of General Pathology, University Hospital Heidelberg, Heidelberg, Germany; 4 Department of Neurology, University Hospital and University of Zuerich, Zuerich, Switzerland; 5 Institute for Neuropathology, Heinrich Heine University Duesseldorf, Duesseldorf, Germany; 6 German Cancer Consortium (DKTK), partner site Essen/Duesseldorf, German Cancer Research Center (DKFZ), Heidelberg, Germany; 7 Department of Neuropathology of the University of Bonn, Bonn, Germany; 8 Institute for Neuropathology of the University of Essen, Essen, Germany; 9 Institute of Neuropathology, Medical Faculty, University of Freiburg, Freiburg, Germany; 10 Signalling Research Centres BIOSS and CIBSS, University of Freiburg, Freiburg, Germany; 11 Center for Basics in NeuroModulation (NeuroModulBasics), Faculty of Medicine, University of Freiburg, Freiburg, Germany; 12 Berta-Ottenstein-Programme for Advanced Clinician Scientists, Faculty of Medicine, University of Freiburg, Freiburg, Germany; 13 Institute of Neuropathology, University of Giessen, Giessen, Germany; 14 Department for Neuropathology, Institute for Pathology, Hannover Medical School, Hannover, Germany; 15 Institute of Neuropathology, University Hospital Muenster, Muenster, Germany; 16 Institute for Pathology and Neuropathology of the University of Tuebingen, Comprehensive Cancer Center Tuebingen, Tuebingen, Germany; 17 Institute for Pathology, Sozialstiftung Bamberg, Bamberg, Germany; 18 Institute for Neuropathology of the Friedrich-Alexander University of Erlangen-Nuernberg (FAU), Erlangen, Germany; 19 Department of Neuropathology, University Hospital of Cologne, Cologne, Germany; 20 Institute for Neuropathology of the University of Magdeburg, Magdeburg, Germany; 21 Institute of Pathology, University of Bern, Bern, Switzerland; 22 Department of Cellular Pathology, Queen’s Hospital, Romford, UK; 23 Department of Molecular Neuroscience, UCL Queen Square Institute of Neurology, Queen Square, London, UK; 24 Department of Neurodegenerative Disease, UCL Queen Square Institute of Neurology, Queen Square, London, UK; 25 Clinic for Neurosurgery, University Hospital Heidelberg, Heidelberg, Germany; 26 Clinic for Neurosurgery, University of Mannheim, Mannheim, Germany; 27 Department of Neurology, Medical Faculty Mannheim, Heidelberg University, Mannheim, Germany; 28 Clinical Cooperation Unit Neuroimmunology and Brain Tumor Immunology, German Cancer Research Center (DKFZ), Heidelberg, Germany; 29 Division of Pediatric Neurooncology, German Cancer Research Center (DKFZ), Heidelberg, Germany; 30 Department of Pediatric Oncology and Hematology, University Hospital Heidelberg, Heidelberg, Germany; 31 Hopp Children’s Cancer Center Heidelberg (KiTZ), 69120 Heidelberg, Germany; 32 Neurology Clinic, University of Heidelberg Medical Center, Heidelberg, Germany; 33 Division of Experimental Neurosurgery, Department of Neurosurgery, Ruprecht-Karls-University Heidelberg, Heidelberg, Germany; 34 Pediatric Glioma Research Group, German Cancer Research Center (DKFZ), 69120 Heidelberg, Germany; 35 Department of Neuropathology, Charité – Universitaetsmedizin Berlin, corporate member of Freie Universitaet Berlin, Humboldt-Universitaet zu Berlin, and Berlin Institute of Health, Berlin, Germany; 36 German Cancer Consortium (DKTK), Partner Site Berlin, German Cancer Research Center (DKFZ), Heidelberg, Germany

**Keywords:** Cerebellar glioblastoma, Methylation-based classification, Copy number variation load, Anaplastic pilocytic astrocytoma, Anaplastic astrocytoma with piloid features, Integrated diagnosis

## Abstract

In this multi-institutional study we compiled a retrospective cohort of 86 posterior fossa tumors having received the diagnosis of cerebellar glioblastoma (cGBM). All tumors were reviewed histologically and subjected to array-based methylation analysis followed by algorithm-based classification into distinct methylation classes (MCs). The single MC containing the largest proportion of 25 tumors diagnosed as cGBM was MC anaplastic astrocytoma with piloid features representing a recently-described molecular tumor entity not yet included in the WHO Classification of Tumours of the Central Nervous System (WHO classification). Twenty-nine tumors molecularly corresponded to either of 6 methylation subclasses subsumed in the MC family GBM IDH wildtype. Further we identified 6 tumors belonging to the MC diffuse midline glioma H3 K27 M mutant and 6 tumors allotted to the MC IDH mutant glioma subclass astrocytoma. Two tumors were classified as MC pilocytic astrocytoma of the posterior fossa, one as MC CNS high grade neuroepithelial tumor with BCOR alteration and one as MC control tissue, inflammatory tumor microenvironment. The methylation profiles of 16 tumors could not clearly be assigned to one distinct MC. In comparison to supratentorial localization, the MC GBM IDH wildtype subclass midline was overrepresented, whereas the MCs GBM IDH wildtype subclass mesenchymal and subclass RTK II were underrepresented in the cerebellum. Based on the integration of molecular and histological findings all tumors received an integrated diagnosis in line with the WHO classification 2016. In conclusion, cGBM does not represent a molecularly uniform tumor entity, but rather comprises different brain tumor entities with diverse prognosis and therapeutic options. Distinction of these molecular tumor classes requires molecular analysis. More than 30% of tumors diagnosed as cGBM belong to the recently described molecular entity of anaplastic astrocytoma with piloid features.

**Electronic supplementary material:**

The online version of this article (10.1186/s40478-019-0801-8) contains supplementary material, which is available to authorized users.

## Introduction

Glioblastoma (GBM) of cerebellar localization (cGBM) constitutes less than 1% of all GBMs [1, 16]. Morphological distinction from other established glioma entities of the posterior fossa is often challenging. Particularly difficult is the histological separation from the recently described anaplastic astrocytomas with piloid features (AAP) which exhibit a more favorable clinical outcome compared to GBM IDH wt and which have been shown to harbor MAPK pathway alterations as potential therapeutic targets [25]. GBMs can be divided into molecular subgroups based on their epigenetic profiles [7, 33]. Most frequent are the MCs GBM RTK II and MC GBM mesenchymal (MES) followed by MC GBM RTK I, MC GBM RTK III, MC GBM midline (MID), MC GBM MYCN and MC GBM H3 G34 mutant (G34) [31]. The WHO classification currently does not discriminate between these subgroups and collectively classes them as GBM IDH wildtype (wt). Several studies and case reports have shown that patients with cGBM are younger at first diagnosis than patients with supratentorial GBM (sGBM) [2, 10, 16, 37]. So far, only single investigations on genetic and epigenetic profiles of cGBMs have been carried out. Mutations and DNA copy number changes commonly observed in cerebral malignant gliomas were less frequently encountered infratentorially. Moreover, enrichment for *PDGFRA* and *ATRX* alterations was found, whereas *EGFR* and *TERT* alterations were rare [9, 13, 21, 36]. Two previous studies on methylation profiles of cGBMs have reported assignment to the MCs diffuse midline glioma H3 K27 M mutant (DMG K27), GBM RTK I, GBM MID and IDH mutant glioma subclass astrocytoma (A IDH). However, the inclusion of only 14 and 4 cGBMs in these studies is a limitation for general conclusions [9, 23]. Further, the MCs AAP and GBM MID were not represented in the reference sets of the respective clustering analyses. In summary, molecular markers and epigenetic profiles of cGBMs have not yet been comprehensively evaluated. Therefore, definition of clinical and molecular features warranting the designation as a distinct GBM variant is still controversially discussed [5, 9, 13].

With this work we set out to molecularly characterize cGBM by applying a more comprehensive molecular diagnostic work-up.

## Materials and methods

### Sample selection

We collected formalin fixed and paraffin embedded (FFPE) tissue from 86 patients with cerebellar tumors having received the diagnosis of GBM according to the WHO classification 2007 [20]. The tumor samples were collected and originally diagnosed at neuropathological institutions of the universities of Bern, Bonn, Dresden, Duesseldorf, Erlangen, Essen, Freiburg, Marburg/Giessen, Hannover, Heidelberg, Cologne, London, Magdeburg, Miami, Moscow, Muenster, Romford, Tuebingen and Zurich. We also obtained tumors via the German Glioma Network that had been centrally reviewed at the German Brain Tumor Reference Center in Bonn. Tumors extending beyond the posterior fossa were included only if the major tumor portion was within the cerebellum and if the clinical data supported a primarily cerebellar origin. Tumors with obvious initial manifestation in the brain stem prompting the diagnosis of malignant brain stem glioma and tumors with known additional supratentorial manifestation were excluded. Tissue collection and processing as well as data collection were in compliance with local ethics regulations and approval. Upon identification of a suitable area on HE sections DNA was extracted employing standard methods as previously described [25].

For each tumor, the following data sets were collected, if available: local histological diagnosis, patient gender, patient age at histological diagnosis of GBM, tumor localization and information on the time point of tissue sampling (primary surgery versus re-resection). For comparison of cGBM and sGBM cohorts two-sided T-test was applied in Excel.

### Histology and immunohistochemistry

Morphological criteria for diagnosing GBM were the appearance of a malignant glial tumor with astrocytic differentiation, brisk mitotic activity and the presence of necrosis and/or prominent microvascular proliferation [20]. Routine immunohistochemical analyses included assessment of IDH1 R132H by the H09 antibody (Dianova, Hamburg, Germany), of BRAF V600E by the VE1 antibody (Roche, Basel, Switzerland), of ATRX (Sigma-Aldrich, St. Louis, Missouri, USA) and of H3 K27 M (Merck Millipore, Burlington, Massachusetts, USA) status. Immunohistochemistry was performed on a Ventana BenchMark XT Immunostainer (Ventana Medical Systems, Tucson, Arizona, USA) using established protocols. For dilutions and antibody details, see Additional file 1. Immunostaining with antibodies against BRAF V600E, IDH1 R132H and H3 K27 M was scored as either positive or negative. Care was taken to exclude unspecific binding and binding to non-tumorous cells. Loss of nuclear ATRX expression was scored as specific, if tumor cell nuclei showed loss of expression, while nuclei of non-neoplastic cells, such as endothelia, microglia, lymphocytes and reactive astrocytes, were positive. Of note, weak to moderate staining of tumor cell cytoplasm was occasionally seen and was considered as non-specific [27].

### Methylation-based classification, determination of copy number variations (CNVs) and statistics

DNA was extracted from FFPE tissue using the automated Maxwell system (Promega, Fitchburg, Massachusetts, USA) according to the manufacturer’s instructions. DNA concentration was determined using the Qubit dsDNA BR Assay kit (Invitrogen, Carlsbad, California, USA) following the producer’s guidelines. DNA was subjected to methylation analysis applying Illumina 450 K BeadChip (84/86 samples) or EPIC analysis (2/86 samples) (Illumina, Carlsbad, California, USA) as previously described [25]. IDAT files were analyzed by a recently described algorithm designated brain tumor classifier [7] (www.molecularneuropathology.org). Using this brain tumor classifier highly characteristic methylation classes (MCs) were established, for which correlations to the respective brain tumor entities in the WHO classification were evident. Classifier scores with a probability greater 0.9 were taken as indicative for the respective MC. CNVs were calculated from the IDAT files using the R/Bioconductor package conumee [14] (http://bioconductor.org/packages/release/bioc/html/conumee.html) after additional baseline correction (https://github.com/dstichel/conumee). As a measure for the frequency of structural chromosomal aberrations in a tumor DNA sample the CNV load was computed [30]. It represents the cumulative length of all aneuploid chromosomal segments of a sample. Amplifications (amp) and homozygous deletions (del) were visually assessed from the CNV plots and were defined as focal regions of copy number gain or loss with a notably higher amplitude than regions of suspected single-copy gains or losses. IDAT files were also employed for t-SNE and unsupervised clustering analyses. Tumors grouping together in these analyses were designated as methylation clusters.

The DNA methylation array data were processed with the R/Bioconductor package minfi (version 1.20) [22]. For unsupervised hierarchical clustering analysis of cGBMs and reference samples, we selected the 20,000 most variably methylated CpG sites across the dataset according to median absolute deviation. Pairwise similarity of samples was calculated using Euclidean distance. Clusters were then linked according to the Ward’s linkage method. The t-SNE plot was computed via the R package Rtsne [23] using the 20,000 most variable CpG sites according to standard deviation, 3000 iterations and a perplexity value of 10.

### *H3F3A, BRAF, IDH1, IDH2* and *TERT* promoter mutation analysis and next generation sequencing

Mutation analyses were performed by bidirectional Sanger sequencing as previously described [17, 25]. 27/86 tumors (31%) have been examined by next generation sequencing employing a gene panel also covering the *TERT* promoter [28].

### Assignment of integrated diagnoses

An integrated diagnosis was assigned to each tumor applying the procedures introduced by the WHO classification 2016 [19].

### Reference datasets for t-SNE and summary-CNV profiles

For t-SNE, clustering analysis and calculation of summary-CNV profiles, the following 12 glioma reference MCs were used comprising a sum of 707 reference cases: diffuse midline glioma H3 K27 M mutant (DMG K27, 38 cases), GBM IDH wt H3 G34 mutant (GBM G34, 37 cases), GBM IDH wt subclass midline (GBM MID, 23 cases), GBM IDH wt subclass mesenchymal (GBM MES, 128 cases), GBM IDH wt subclass RTK I (GBM RTK I, 72 cases), GBM IDH wt subclass RTK II (GBM RTK II, 171 cases), GBM IDH wt subclass RTK III (GBM RTK III, 9 cases), GBM IDH wt subclass MYCN (GBM MYCN, 18 cases), low grade glioma subclass posterior fossa pilocytic astrocytoma (PA PF, 114 cases), CNS high grade neuroepithelial tumor with BCOR alteration (HGNET BCOR, 22 cases) anaplastic astrocytoma with piloid features (AAP, 41 cases) and IDH mutant glioma subclass astrocytoma (A IDH, 34 cases). Notably, MC A IDH does not distinguish between IDH mutant astrocytoma and IDH mutant glioblastoma. Detailed descriptions of the reference MCs used in this study are outlined under: https://www.molecularneuropathology.org.

## Results and discussion

### Patients with cGBM present at a younger age than patients with sGBM

Within the cohort of 86 patients with cGBM, 73 patients were adults with a median age of 56 years comparing well with median ages ranging from 50 to 58 years reported in previous studies on adult patients with cGBM, respectively [1, 37]. Median age of all 86 patients with cGBM was 52 years (range: 5–88 years). In contrast, the median age of our reference cohort of patients with sGBMs was 60 years (range: 0–86 years) which is in line with previous reports [35]. Student’s t-Test comparing the ages of our patients with supratentorial compared to cGBM revealed a *p* value of 0.004 confirming that cGBM patients present at a younger age at diagnosis than patients with sGBM.

### The majority of cGBMs correnponds to the methylation clusters AAP and GBM MID

**Fig. 1:**
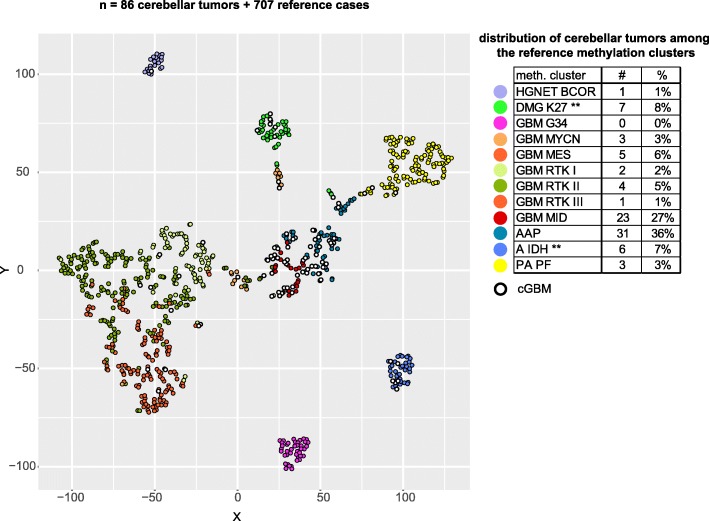
Methylation-based t-SNE distribution of 86 tumors designated cerebellar glioblastoma and 12 established reference methylation clusters comprising a reference cohort of 707 gliomas. Reference cases are indicated as colored dots with each color representing one reference methylation cluster. Tumors of the study cohort are indicated as black-rimmed circles. The table shows the distribution of tumors in the cerebellar study cohort among the reference methylation clusters. cGBM – cerebellar glioblastoma, meth. Cluster – methylation cluster, # - number of tumors in the cGBM cohort, HGNET BCOR – high grade neuroepithelial tumor with BCOR alteration, DMG K27 – diffuse midline glioma H3 K27 M mutant, GBM G34 – glioblastoma IDH wildtype subclass H3 G34 mutant, GBM MYCN – glioblastoma IDH wildtype subclass MYCN, GBM MES – glioblastoma IDH wildtype subclass MES, GBM RTK I/II/III – glioblastoma IDH wildtype subclass(es) RTK I/II/III, GBM MID – glioblastoma IDH wildtype subclass midline, AAP – anaplastic astrocytoma with piloid features, A IDH – IDH mutant glioma, PA PF – low grade glioma subclass posterior fossa pilocytic astrocytoma. ** Frequencies of these tumors may be biased depending on the supplier diagnosis, the date of diagnosis and the availability and application of antibodies or sequencing methods detecting *IDH* and histone mutations

t-SNE distribution and unsupervised hierarchical clustering analysis showed that more than half of the histologically diagnosed cGBMs (54/86 tumors, 63%) fall into the methylation clusters AAP and GBM MID (Fig. 1, Additional file 2). Notably, the methylation profiles of these two entities show close proximity to each other. The methylation cluster GBM MID comprises tumors with the histological diagnosis of glioblastoma and location in midline structures (thalamus, cerebellum, spinal cord). These tumors share epigenetic similarities with the methylation cluster DMG K27, but lack the characteristic histone mutation [26]. The second largest group of cerebellar tumors was allotted to the methylation clusters GBM RTK I/II and MES, thus corresponding to typical profiles of supratentorial GBMs IDH wt. A small fraction was allocated to the methylation cluster DMG K27, whereas another small fraction was allotted to the methylation cluster A IDH. Finally, only single cases mapped to the methylation clusters HGNET BCOR, GBM MYCN and PA PF. Some of these tumors occurred in pediatric patients (see Additional file 3a).

The close proximity of the cerebellum to the brainstem may explain the inclusion of 8 *H3 K27 M* mutant tumors into our cohort receiving the integrated diagnosis DMG K27 WHO grade IV although our inclusion algorithm excluded tumors with obvious localization in the brainstem or pons [4, 33]. However, inclusion of *H3 K27 M* mutant tumors into a series of cerebellar gliomas without brain stem manifestation has also been reported in previous studies [11, 13, 23].

In a recent publication, the methylation profile of one of four adult cGBMs was classified as MC A IDH, whereas the three others were allotted to MC GBM MID [9]. So far, *IDH* mutations have been known to occur preferentially in gliomas of supratentorial localization, whereas little is known about their frequency in infratentorial diffuse gliomas [33]. One investigation of *IDH1* R132H mutations in gliomas of the infratentorial compartment revealed an overall fraction of 7%. However, in that study *IDH* mutations were exclusively found in tumors of the brainstem, but in none of 12 cerebellar tumors [12]. Further analyses of larger cohorts of infratentorial gliomas are necessary to determine the specific *IDH* mutation frequencies in the subcompartments spinal cord, brainstem and cerebellum.

### Different distribution of MCs in cerebellar versus supratentorial tumors

In comparison to supratentorial sites, MC GBM MID was overrepresented, MCs GBM RTK II and GBM MES were underrepresented, whereas MC GBM G34 was not represented at all in the cerebellum (Table 1). A slight cerebellar enrichment appeared to occur for the MCs DMG K27 and A IDH. An increased number of DMG in the cerebellum can be expected, because occurrence in older patients not exhibiting the characteristic localization restricted to brain stem and pons has been described [6, 22, 29]. However, the high proportion of GBM IDH mutant (GBM IDH mut) was not expected. It should be considered that reported frequencies of DMG K27 and GBM IDH mut in cGBM cohorts may be biased depending on the date of diagnosis, the availability and application of antibodies and sequencing methods detecting *IDH* and histone *H3* mutations.

**Table 1:** Methylation (sub)class^a^ distribution of cerebellar and supratentorial WHO grade IV gliomas

methylation (sub)class^a^	supratentorial	Percent	cerebellar	Percent
	*n* = 522		*n* = 42	
GBM G34	37	7	0	0
GBM MES	127	24	4	10
GBM MID	23	4	11	26
GBM MYCN	18	3	3	7
GBM RTK I	72	14	8	19
GBM RTK II	171	33	2	5
GBM RTK III	9	2	1	2
DMG K27^b^	33	6	7	17
A IDH^b^	32	6	6	14

*GBM IDH mut* glioblastoma IDH mutant, *DMG K27* diffuse midline glioma H3 K27 M mutant, *GBM G34* glioblastoma IDH wildtype subclass H3 G34 mutant, *GBM MYCN* glioblastoma IDH wildtype subclass MYCN, *GBM MES* glioblastoma IDH wildtype subclass MES, *GBM RTK I/II/III* glioblastoma IDH wildtype subclass(es) RTK I/III/III, *GBM MID* glioblastoma IDH wildtype subclass midline, *A IDH* IDH mutant glioma subclass astrocytoma or high grade astrocytoma

^a^ Methylation class is defined as group of tumors with the same classifier diagnosis (= epigenetic subgroup) which was allotted to a tumor sample with a classifier score above 0.9

^b^ Frequencies of these tumors may be biased depending on the supplier diagnosis, the date of diagnosis and the availability and application of antibodies or sequencing methods detecting *IDH* and histone mutations

### Integrated diagnoses of the 86 investigated cerebellar tumors

Based on morphology, methylation profile and single molecular parameters an integrated diagnosis was assigned in line with the recommendations in the WHO classification 2016 update [19]. Additional file 3a provides an overview of integrated diagnoses, clinical and molecular data of all 86 cerebellar tumors. GBM morphology and matching methylation subclasses of GBM IDH wt occurred in 29 cases prompting the integrated diagnosis of GBM IDH wt WHO grade IV. In 8 additional tumors (cases 1–8) we also provided the integrated diagnosis of GBM IDH wt WHO grade IV for reasons given in Additional file 3a. It should be kept in mind that the integrated diagnosis GBM IDH wt comprises 7 different MCs [7, 8, 31], (https://www.molecularneuropathology.org).

Because AAP lacks unifying morphological criteria, however, is characterized by a distinct methylation profile, occurrence of MC AAP (calibrated score > 0.9) was taken as evidence for this diagnosis in a total of 25 cases. Two additional tumors (cases 16 and 28 in Additional file 3a) with high, but below-threshold classifier scores for the MC AAP received this diagnosis because of exhibiting other typical features such as homozygous deletion of *CDKN2A/B* or nuclear loss of ATRX expression [7, 25]. 8 tumors carried the *H3 K27 M* mutation and consequently received the integrated diagnosis of DMG K27 WHO grade IV with 6 of them belonging to the MC DMG K27. Among the remaining two cases (cases 42 and 46 in Additional file 3a) one had a low classifier score for MC DMG K27 and the other received the highest classifier score for MC control tissue, inflammatory tumor microenvironment. However, in both cases tumor cell content was high and prominent inflammatory infiltration was absent. 6 tumors classified as MC A IDH carried an *IDH* mutation and were diagnosed as GBM IDH mut WHO grade IV accordingly. Notably, 5 of the 6 GBMs IDH mut harbored a rare *IDH1* mutation. Inability of the IDH1 R132H antibody in recognizing rare *IDH* mutations likely explains why these tumors initially have not been identified as *IDH* mutant. Whether rare *IDH* mutations are more frequent in the cerebellum cannot be addressed with our series. Further analyses with unbiased and higher patient numbers will be needed for clarification.

Two tumors of MC PA PF, one of them with a *KIAA1549-BRAF* fusion, were re-diagnosed as pilocytic astrocytoma WHO grade I (PA I). One tumor with MC HGNET BCOR and lack of gross chromosomal alterations was diagnosed accordingly. The MC HGNET BCOR has emerged from the analysis of a large cohort of tumors previously termed CNS PNET, presents with characteristic features, but is not yet represented in the WHO classification [32].

Five of the tumors (cases 9–13 in Additional file 3a) were descriptively diagnosed as “tumor NOS” in two cases due to lack of histological slides and as “malignant neuroectodermal brain tumor” in three cases due to lack of diagnostic molecular evidence. Of note, all tumors with inconclusive molecular results (cases 1–13 in Additional file 3a) were dispersed among the reference methylation clusters with the majority falling into the clusters GBM MID and AAP. Additional file 3b depicts the assignment of these tumors in the original t-SNE plot (see also Fig. 1).

**Fig. 2:**
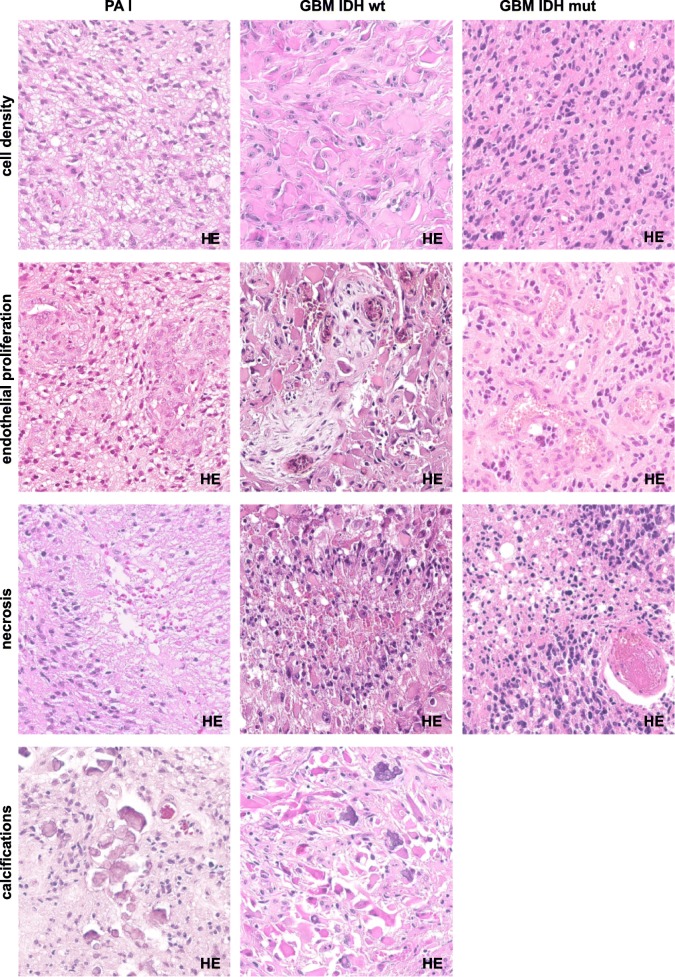
Histological features of five tumors with different integrated diagnoses, but initially designated cerebellar glioblastoma by morphology: HEs of pilocytic astrocytoma (PA I), glioblastoma IDH wildtype (GBM IDH wt) and glioblastoma IDH mutant (GBM IDH mut). All three tumors display a high cell density, vascular endothelial cell proliferation and necrosis. PA I as well as GBM IDH wt even show calcifications. Gamma settings have been adjusted in the second row, column I and II (400-fold magnification)

**Fig. 3:**
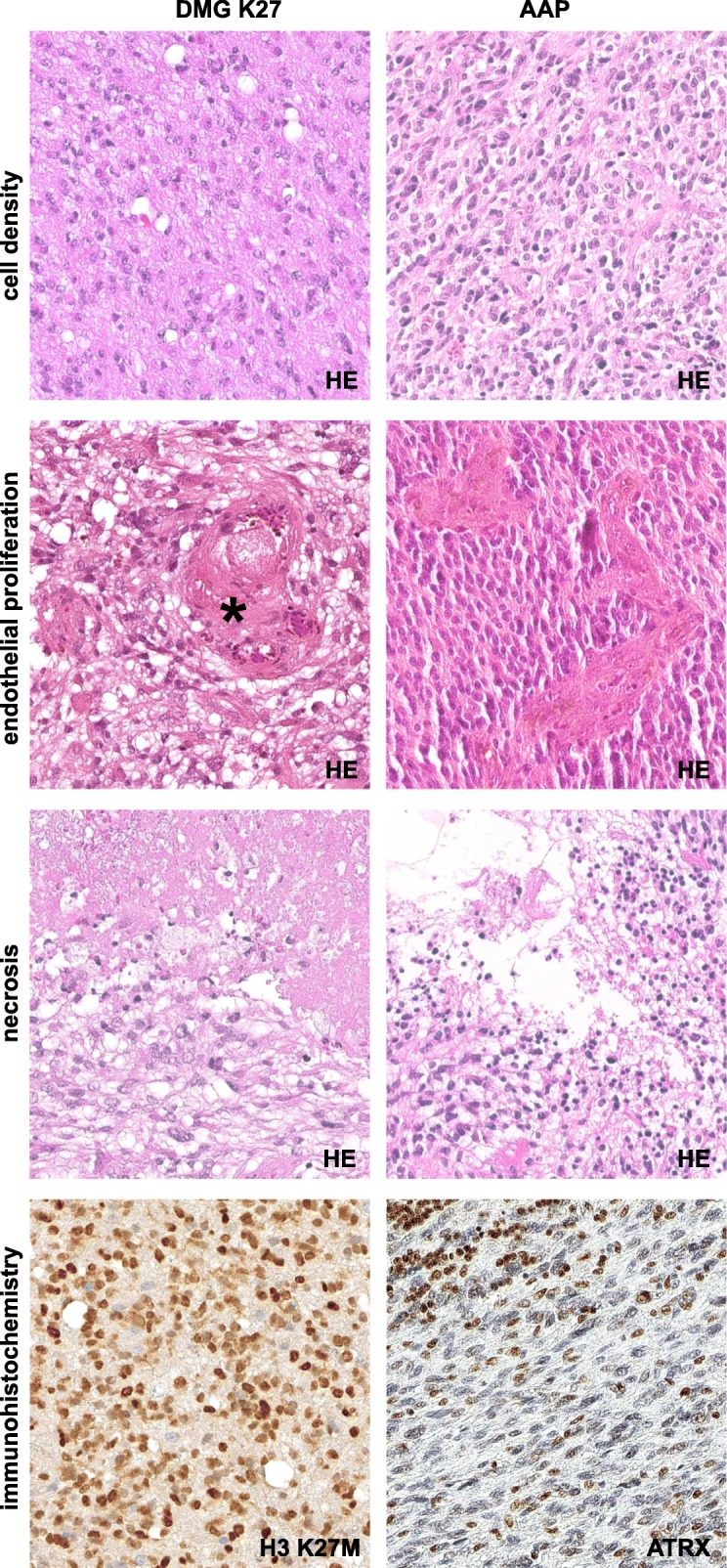
Histological features of five tumors with different integrated diagnoses, but initially designated cerebellar glioblastoma by morphology: HEs of diffuse midline glioma H3 K27M mutant (DMG K27) and anaplastic astrocytoma with piloid features (AAP) also demonstrate a high cell density, vascular endothelial cell proliferation and necrosis. The asterisk marks a thrombosed and re-canalized vessel. H3 K27M mutant protein was found in the DMG K27, whereas nuclear loss of ATRX expression could be observed in the AAP. Gamma settings have been adjusted in the second and fourth row, column I and II. (400-fold magnification)

The fact that only 51/86 (59%) cerebellar tumors histologically diagnosed as cGBM received an integrated diagnosis of a WHO grade IV glioma corresponding to either GBM or DMG K27 (see Additional file 3a) discloses that the morphological diagnosis of cGBM is challenging. Figures 2 and 3 illustrate how five cerebellar gliomas with GBM morphology were given five different integrated diagnoses after performing molecular analysis. These issues with histological differential diagnosis are due to morphological overlaps between entities that have to be considered in case of cerebellar glioma. Histology of AAP for example shows overlaps with GBM, PA I and the variant of pilocytic astrocytoma with anaplastic features (APA). Whilst the diagnosis of APA is made on histological grounds only [19], AAP is a molecularly defined entity which shares histological features with APA, but whose morphological spectrum has been shown to be much more variable [25]. In particular, the presence of necrosis, vascular endothelial proliferation and frequent mitoses, but also the absence of eosinophilic granular bodies or Rosenthal fibers can present a challenge in differentiating these tumors from GBM. Nevertheless, the recognition of AAP is relevant because survival of patients with these tumors has been found to be more favorable than that for patients with GBM IDH wt. In fact, based on current knowledge survival of patients with AAP appears to be comparable to that of patients with GBM IDH mut. In addition, AAPs carry MAPK alterations which may represent therapeutic targets [25]. Tumors diagnosed as AAP and/or APA have been reported to frequently carry *ATRX* alterations [24, 25], which was also evident for the tumors in our series (see Additional file 3a). In a recent study, molecular analyses on 19 cerebellar tumors diagnosed as GBM also revealed 4 cases with *ATRX* alteration with three of them lacking *IDH1* R132H or *H3F3A* K27 M [9]. As these tumors may likely represent AAPs comprehensive methylation analysis would be of interest.

### No significant differences in CNV loads and summary CNV profiles of cerebellar versus supratentorial MCs

**Fig. 4:**
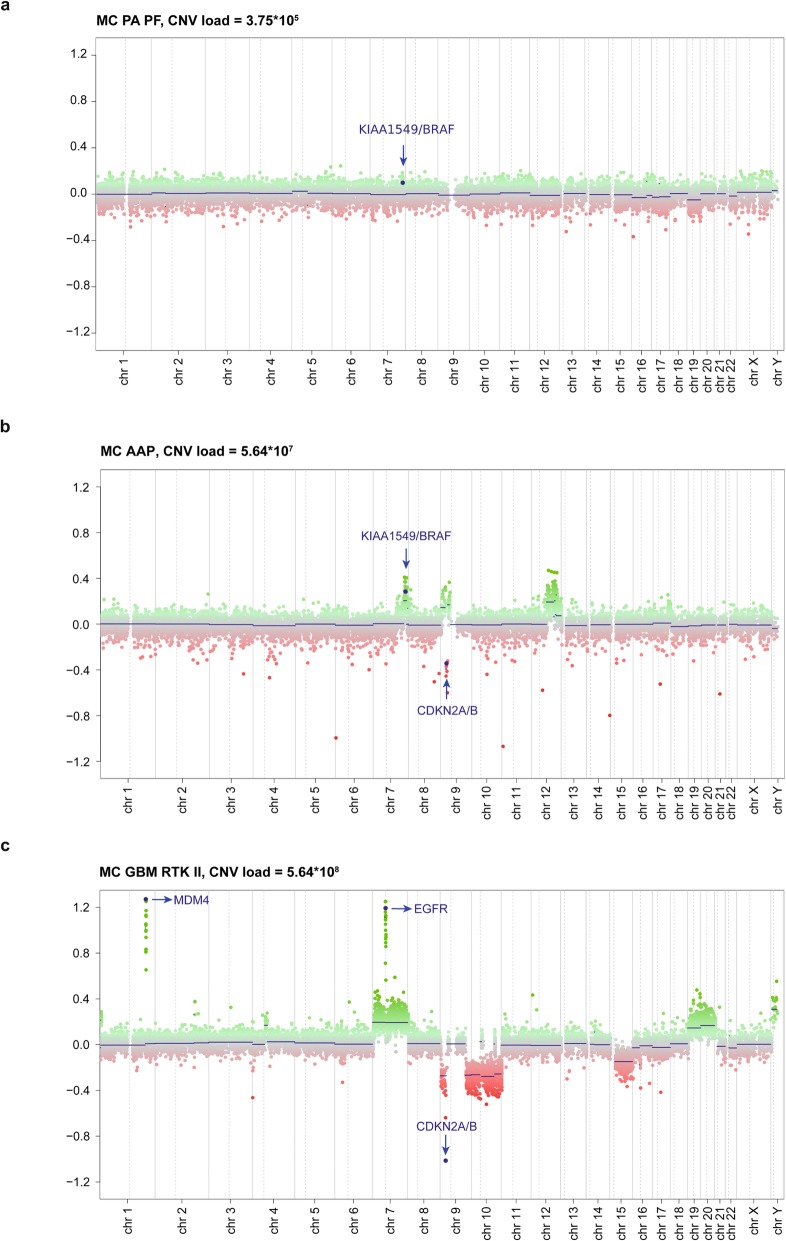
Examples for copy number profiles of three cerebellar gliomas of different methylation classes and accordingly varying copy number variation loads (CNV loads): **a** methylation class low grade glioma subclass posterior fossa pilocytic astrocytoma (MC PA PF) with *BRAF* fusion and comparatively low CNV load; **b** methylation class anaplastic astrocytoma with piloid features (MC AAP) with *BRAF* fusion, *CDKN2A/B* deletion and higher CNV load; **c** methylation class glioblastoma IDH wildtype subclass RTK II (MC GBM RTK II) with *CDKN2A/B* deletion, *EGFR* and *MDM4* amplification, combined chromosome 7 gain and chromosome 10 loss and highest CNV load

Average CNV loads were calculated for the MCs of cerebellar tumors and compared to the MCs of supratentorial reference cases (Additional file 4). No obvious differences were observed except for a lower CNV load in cerebellar tumors of the MC A IDH compared to tumors belonging to the supratentorial control MC A IDH. As the number of cerebellar IDH-mutant tumors in this cohort was limited, further analyses of a larger cohort are necessary for confirmation. Moreover, a trend towards a higher CNV load in cerebellar MC GBM MID tumors compared to supratentorial MC GBM MID tumors was observed. In Fig. 4, individual CNV profiles of three cerebellar tumors histologically diagnosed as GBM, but resolving into the MC PA PF, MC AAP and MC GBM RTK I are shown. The respective CNV loads are indicated suggesting an association of CNV load with malignancy [30]. We further compared summary CNV plots of cerebellar and supratentorial MCs as illustrated in Additional file 5. For most methylation subclasses larger chromosomal aberrations equally occurred in both cerebellar and supratentorial localization. One exception was the MC GBM RTK I where chromosome 7 gain was less frequently observed in the cerebellar tumors. In contrast, cerebellar tumors of the MC A IDH appeared to show a higher frequency of chromosome 7 gain compared to their supratentorial counterparts. Because the case number for cerebellar MCs was low, these data require further confirmation.

### *PDGFRA* amp and *CDKN2A/B* loss are overrepresented, whereas *EGFR* amp is underrepresented in cerebellar versus supratentorial tumors of the MC family GBM IDH wt

In addition to summary CNV profiles assessing larger chromosomal aberrations, focal alterations comprising amplifications and deletions were analyzed. Recent studies proposed that case cohorts of cGBMs are enriched for *PDGFRA* alterations and *CDKN2A/B* loss, whereas *EGFR* amp is comparatively rare [9, 23, 34, 36]. We assessed the frequencies of these alterations in 29 cerebellar tumors of the MC family GBM IDH wt versus 457 supratentorial reference tumors (see Additional files 3 and 6) and also considered their distribution differences between the individual methylation subclasses. Indeed, *PDGFRA* amp and *CDKN2A/B* loss were more frequently observed among the cerebellar compared to supratentorial tumors. High proportions of these alterations were particularly evident for the MCs GBM MID and RTK I. The fact that these MCs were more frequent among the cerebellar (19/29, 65%) compared to supratentorial tumors (95/457, 21%) may explain the enrichment of *PDGFRA* amp and *CDKN2A/B* loss previously reported in cGBMs. Our data also confirmed that *EGFR* amp is less frequently encountered in cGBMs. This alteration is most abundant in the MCs GBM RTK II and GBM MES, whereas not found in the MC GBM MID. Therefore, the different distributions of these MCs in the cerebellum also appear to explain the lower frequency of *EGFR* amp found in previous studies on cGBMs.

### *TERT* promoter mutations occur less frequently in cerebellar than in supratentorial tumors of the MC family GBM IDH wt

*TERT* promoter mutations have been reported in 54 to 84% of primary GBMs [3, 15, 17, 18, 26]. One previous study identified a *TERT* promoter mutation in only one of 27 diffuse cerebellar gliomas. Another investigation revealed this alteration in two of 19 adult cGBMs [9, 23]. In our series, *TERT* promoter mutations were detected in 31% (9/29) of cerebellar compared to 77% (98/127) of supratentorial tumors of the MC family GBM IDH wt (see Additional files 3 and 6). Interestingly, none of the tumors allotted to the MC GBM MID harbored such an alteration, which has already been reported previously [26]. The vast majority of *TERT* promoter mutations was distributed among the MCs GBM MES, RTK I and RTK II. Therefore, overrepresentation of the MC GBM MID, but also underrepresentation of the MCs GBM MES and GBM RTK II may have contributed to the comparatively low frequency of *TERT* promoter mutations among cerebellar tumors of the MC family GBM IDH wt.

## Conclusions

Molecular analysis of a series of 86 histologically classified GBMs of the cerebellum revealed that this tumor group contains clinically and genetically different tumor entities. The frequencies of molecular subclasses differ between cerebellar and supratentorial localization. To only 37/86 tumors (43%) the integrated diagnosis of GBM IDH wt was assigned. The most important entity to separate from the mixed bag of tumors diagnosed as cGBM is the recently described AAP (27/86 tumors, 31%). Assignment of this diagnosis is relevant as patients with AAP have a more favorable clinical outcome compared to GBM IDH wt and their tumors may harbor a targetable MAPK alteration.

## Additional files

Additional file 1: Primary antibodies used for immunohistochemistry. (PDF 52 kb)

Additional file 2: Methylation-based clustering analysis of 86 cerebellar glioblastomas (cGBMs) with 707 reference cases. (PDF 16356 kb)

Additional file 3: **a**. Molecular and clinical data of all 86 cerebellar gliomas sorted by integrated diagnosis. **b**. mapping of cases 1–13 in the t-SNE (see Fig. 1). (ZIP 890 kb)

Additional file 4: Average CNV loads of cerebellar and reference glioma methylation classes. (PDF 135 kb)

Additional file 5: Summary copy number variation (CNV) profiles. (PDF 1900 kb)

Additional file 6: Frequencies of PDGFRA amplification (amp), EGFR amp, CDKN2A/B loss and TERT promoter mutation in supratentorial GBMs IDH wt. (XLSX 36 kb)

## Data Availability

All processed data generated or analyzed are included in this published article and its supplementary information files.

## Abbreviations

A IDH: IDH mutant glioma subclass astrocytoma
AAP: Anaplastic astrocytoma with piloid features
amp: Amplification
APA: Anaplastic pilocytic astrocytoma
ATRX: Alpha thalassemia/mental retardation syndrome X-linked
BRAF: B-Raf proto-oncogene, serine/threonine kinase
CDKN2A/B: Cyclin dependent kinase inhibitor A/B
cGBM: Cerebellar glioblastoma
CNS: Central nervous system
CNV: Copy number variation
del: Deletion
DMG K27: Diffuse midline glioma H3 K27 M mutant
EGFR: Epidermal growth factor receptor
FFPE: Formalin-fixed, paraffin-embedded
G34: Subclass H3 G34 mutant
GBM: Glioblastoma
H3: Histone H3
HGNET BCOR: CNS high grade neuroepithelial tumor with BCOR alteration
IDH: Isocitrate dehydrogenase
MAPK: Mitogen-activated protein kinase
MC: Methylation class
MES: Subclass mesenchymal
MID: Subclass midline
mut: Mutant
NOS: Not otherwise specified
PA I: Pilocytic astrocytoma WHO grade I
PA PF: Low grade glioma subclass posterior fossa pilocytic astrocytoma
PDGFRA: Platelet derived growth factor receptor alpha
PNET: Primitive neuroectodermal tumor
sGBM: Supratentorial glioblastoma
TERT: Telomerase reverse transcriptase
t-SNE: t-distributed stochastic neighbor embedding
WHO: World Health Organization
wt: Wildtype
