# Supplementary material for: Tumors diagnosed as cerebellar glioblastoma comprise distinct molecular entities
Source: Acta Neuropathol Commun. 2019 Oct 28;7:163. doi: 10.1186/s40478-019-0801-8 (PMC6816155; doi:10.1186/s40478-019-0801-8)
Supplement: Supplementary file 1 — Primary antibodies used for immunohistochemistry. (PDF 52 kb) [file 40478_2019_801_MOESM1_ESM.pdf]

Online resource 1: Primary antibodies used for immunohistochemistry

Antibody	Manufacturer	Clone	Catalogue No.	Pretreatment	Antibody dilution	Antibody incubation
ATRX	Sigma-Aldrich	poly-clonal	HPA001906	CC1, 90 min	1:200	120 min
H3.3 K27M	Merck Millipore	poly-clonal	ABE419	CC1, 64 min	1:500	32 min
IDH1 R132H	Dianova	H09	DIA-H09	CC1, 64 min	1:25	32 min
BRAF V600E	Roche	VE1	790-4855	CC1, 64 min	1:5	32 min

List of primary antibodies used for immunohistochemistry including manufacturer, clone, dilutions and incubation conditions on a Ventana BenchMark XT Immunostainer.

Reinhardt et al., Acta Neuropathologica: "Tumors diagnosed as cerebellar glioblastoma comprise distinct molecular entities ", correspondence: andreas.vondeimling@med.uni-heidelberg.de
