# Supplementary material for: Tumors diagnosed as cerebellar glioblastoma comprise distinct molecular entities
Source: Acta Neuropathol Commun. 2019 Oct 28;7:163. doi: 10.1186/s40478-019-0801-8 (PMC6816155; doi:10.1186/s40478-019-0801-8)
Supplement: Supplementary file 2 — Methylation-based clustering analysis of 86 cerebellar glioblastomas (cGBMs) with 707 reference cases. (PDF 16356 kb) [file 40478_2019_801_MOESM2_ESM.pdf]

Online resource 2: Methylation-based clustering analysis of 86 cerebellar glioblastomas (cGBMs) with 707 reference cases

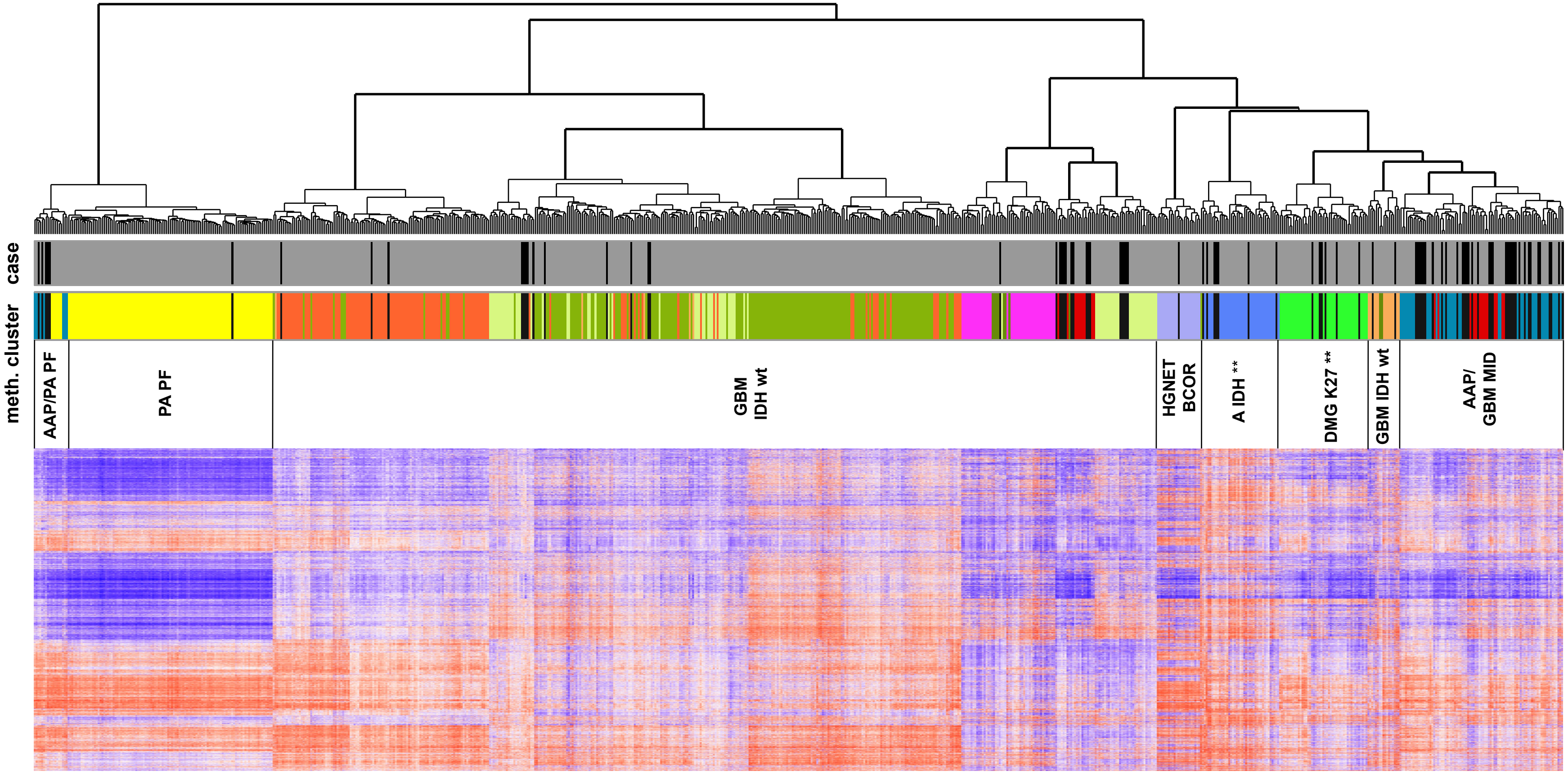

case

- reference cases
- cGBMs

meth. clusters

- PA PF
- HGNET BCOR
- GBM RTK III
- GBM RTK II
- GBM RTK I
- GBM MYCN
- GBM MID
- GBM MES
- DMG K27 **
- GBM G34
- AAP
- A IDH **

distribution of cGBMs among the reference meth. clusters

meth. cluster	#	%
HGNET BCOR	1	1%
DMG K27 **	6	7%
GBM G34	0	0%
GBM MYCN	2	2%
GBM RTK I/II/MES	18	21%
GBM RTK III	1	1%
GBM MID	10	12%
AAP/GBM MID	35	41%
AAP/PA PF	5	6%
A IDH **	7	8%
PA PF	1	1%

Unsupervised methylation-based clustering analysis of 86 tumors histologically diagnosed as cGBMs and 12 established reference methylation clusters comprising a reference cohort of 707 gliomas. Reference cases are indicated in different colors with each color representing one reference methylation cluster. Tumors of the study cohort are indicated in black. The table shows the distribution of cerebellar tumors among the indicated reference methylation clusters. meth. cluster - methylation cluster, GBM IDH wt – glioblastoma IDH wildtype, HGNET BCOR – high grade neuroepithelial tumor with BCOR alteration, DMG K27 – diffuse midline glioma H3 K27M mutant, GBM G34 – glioblastoma IDH wildtype subclass H3 G34 mutant, GBM MYCN – glioblastoma IDH wildtype subclass MYCN, GBM MES – glioblastoma IDH wildtype subclass MES, GBM RTK I/II/III - glioblastoma IDH wildtype subclass(es) RTK I/III/III, GBM MID – glioblastoma IDH wildtype subclass midline, AAP – anaplastic astrocytoma with piloid features, A IDH – IDH mutant glioma, subclass (high grade) astrocytoma, PA PF- low grade glioma subclass posterior fossa pilocytic astrocytoma, # - number of cases in the cGBM cohort.
**Frequencies of these tumors may be biased depending on the supplier diagnosis, the date of diagnosis and the availability and application of antibodies/sequencing methods detecting *IDH* and histone *H3* mutations.

Reinhardt et al., Acta Neuropathologica:
„Tumors diagnosed as cerebellar glioblastoma comprise distinct molecular entities",
correspondence: annekathrin.reinhardt@med.uni-heidelberg.de
