# Supplementary material for: Tumors diagnosed as cerebellar glioblastoma comprise distinct molecular entities
Source: Acta Neuropathol Commun. 2019 Oct 28;7:163. doi: 10.1186/s40478-019-0801-8 (PMC6816155; doi:10.1186/s40478-019-0801-8)
Supplement: Supplementary file 3 — a. Molecular and clinical data of all 86 cerebellar gliomas sorted by integrated diagnosis. b. mapping of cases 1–13 in the t-SNE (see Fig. 1). (ZIP 890 kb) [file 40478_2019_801_MOESM3_ESM.zip › ESM_3b_changed.pdf]

Online resource 3b: mapping of cases 1-13 in the t-SNE (see figure 1)

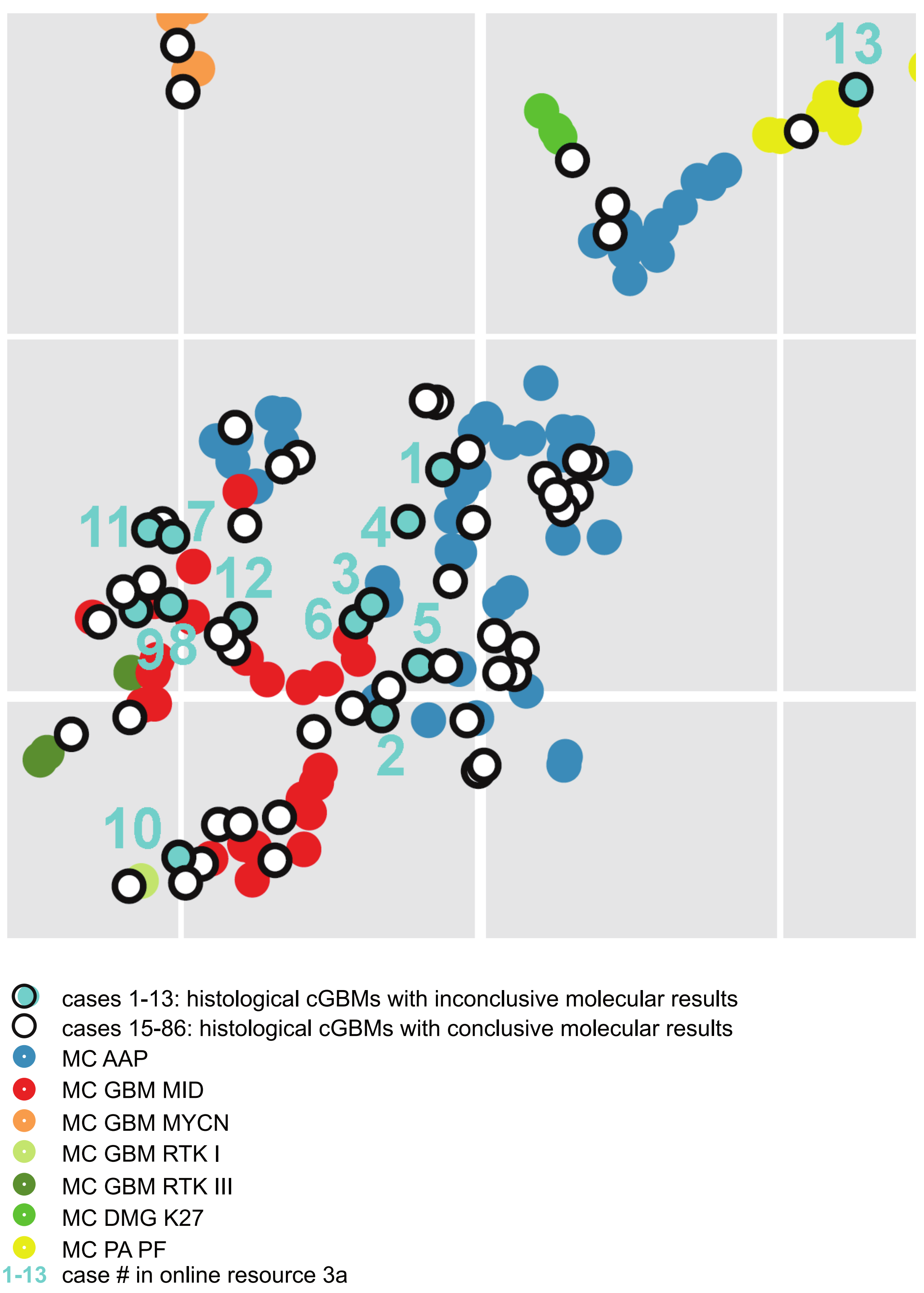

cGBM – cerebellar glioblastoma, DMG K27 – diffuse midline glioma H3 K27M mutant, GBM MYCN – glioblastoma IDH wildtype subclass MYCN, GBM MES – glioblastoma IDH wildtype subclass MES, GBM RTK I/III – glioblastoma IDH wildtype subclass(es) RTK I/III, GBM MID – glioblastoma IDH wildtype subclass midline, AAP – anaplastic astrocytoma with piloid features, PA PF – low grade glioma subclass posterior fossa pilocytic astrocytoma.

Reinhardt et al., Acta Neuropathologica: “Tumors diagnosed as cerebellar glioblastoma comprise distinct molecular entities”,
correspondence: andreas.vondeimling@med.uni-heidelberg.de
