# Supplementary material for: Tumors diagnosed as cerebellar glioblastoma comprise distinct molecular entities
Source: Acta Neuropathol Commun. 2019 Oct 28;7:163. doi: 10.1186/s40478-019-0801-8 (PMC6816155; doi:10.1186/s40478-019-0801-8)
Supplement: Supplementary file 4 — Average CNV loads of cerebellar and reference glioma methylation classes. (PDF 135 kb) [file 40478_2019_801_MOESM4_ESM.pdf]

## Online resource 4: Average CNV loads of cerebellar and reference glioma methylation classes

| methylation class | cerebellar tumors | | reference tumors* | | |
|---|---|---|---|---|---|
| | n | average CNV load | n | average CNV load | p value CNV loads |
| MC GBM MES | 4 | $4.05*10^8$ | 127 | $4.60*10^8$ | 0.727 |
| MC GBM MID | 11 | $1.11*10^9$ | 23 | $6.46*10^8$ | 0.053 |
| MC GBM MYCN | 3 | $1.13*10^9$ | 18 | $1.07*10^9$ | 0.761 |
| MC GBM RTK I | 8 | $6.10*10^8$ | 72 | $7.49*10^8$ | 0.261 |
| MC GBM RTK II | 2 | $4.40*10^8$ | 171 | $6.23*10^8$ | n.a. |
| MC GBM RTK III | 1 | $2,13*10^9$ | 9 | $5.38*10^8$ | n.a. |
| MC DMG K27** | 6 | $6.08*10^8$ | 33 | $5.82*10^8$ | 0.894 |
| MC A IDH** | 6 | $3.22*10^8$ | 32 | $8.11*10^8$ | $5.10*10^{-6}$ |
| MC AAP | 25 | $4.41*10^8$ | 16 | $3.83*10^8$ | 0.571 |
| MC HGNET BCOR | 1 | 0 | 14 | $1.29*10^8$ | n.a. |
| MC PA PF | 2 | $2.30*10^8$ | 109 | $8.35*10^7$ | n.a. |

Methylation classes and average CNV loads of 86 cerebellar tumors with the histological diagnosis of glioblastoma in comparison to 624 reference tumors. CNV – copy number variation, MC - methylation class, A IDH – IDH mutant glioma, AAP – anaplastic astrocytoma with piloid features, HGNET BCOR – high grade neuroepithelial tumor with BCOR alteration, PA PF - low grade glioma, pilocytic astrocytoma GBM MID/MES/RTK I/RTK II/RTK III/MYCN – glioblastoma IDH wildtype subclasses midline/ mesenchymal/RTK I/II/III/MYCN, DMG K27 – diffuse midline glioma H3 K27M mutant

*All reference tumors are of supratentorial localization, except for MC PA PF.

** Frequencies of these tumors in the cerebellum may be biased depending on the supplier diagnosis, the date of diagnosis and the availability and application of antibodies/sequencing methods detecting IDH and histone mutations.

*Reinhardt et al., Acta Neuropathologica: "Tumors diagnosed as cerebellar glioblastoma comprise distinct molecular entities", correspondence: andreas.vondeimling@med.uni-heidelberg.de*
