# Supplementary material for: Tumors diagnosed as cerebellar glioblastoma comprise distinct molecular entities
Source: Acta Neuropathol Commun. 2019 Oct 28;7:163. doi: 10.1186/s40478-019-0801-8 (PMC6816155; doi:10.1186/s40478-019-0801-8)
Supplement: Supplementary file 5 — Summary copy number variation (CNV) profiles. (PDF 1900 kb) [file 40478_2019_801_MOESM5_ESM.pdf]

Online resource 5: Summary copy number variation (CNV) profiles

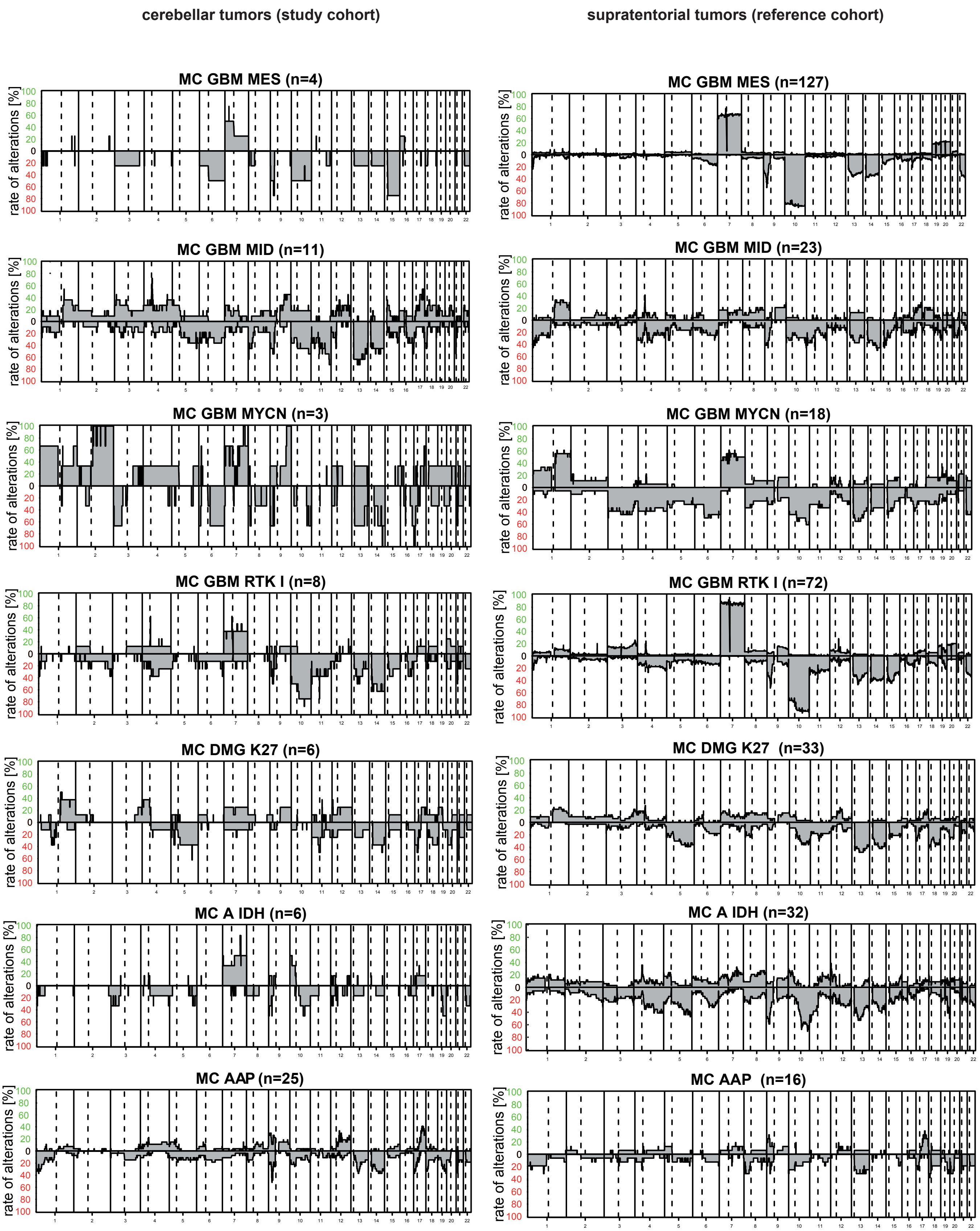

CNV summary plots of 67 cerebellar (cohort) and 366 supratentorial (reference) tumors particularly indicating the percentage of losses and gains seen in the respective methylation classes. MC – methylation class, DMG K27 – diffuse midline glioma H3 K27M mutant, GBM MYCN – glioblastoma IDH wildtype subclass MYCN, GBM MES – glioblastoma IDH wildtype subclass MES, GBM RTK I – glioblastoma IDH wildtype subclass(es) RTK I, GBM MID – glioblastoma IDH wildtype subclass midline, AAP – anaplastic astrocytoma with piloid features, A IDH – IDH mutant glioma.

Reinhardt et al., Acta Neuropathologica: "Tumors diagnosed as cerebellar glioblastoma comprise distinct molecular entities"
correspondence: andreas.vondeimling@med.uni-heidelberg.de
